# Biodegradation of Atrazine by the Novel* Klebsiella variicola* Strain FH-1

**DOI:** 10.1155/2019/4756579

**Published:** 2019-07-28

**Authors:** Jinpeng Zhang, Shuang Liang, Xinhong Wang, Zhongbin Lu, Peng Sun, Hao Zhang, Fengjie Sun

**Affiliations:** ^1^College of Resource and Environment, Jilin Agricultural University, Changchun, China; ^2^Department of Computer Science, Iowa State University, Ames, IA, USA; ^3^School of Science and Technology, Georgia Gwinnett College, Lawrenceville, GA, USA

## Abstract

Bacterial strain FH-1 with high efficiency of degrading Atrazine is separated by means of enrichment culture from the soil applied with Atrazine for many years. FH-1, recognized as* Klebsiella variicola *based on phylogenetic analysis of 16S rDNA sequences, can grow with Atrazine which is the sole nitrogen source. In fluid inorganic salt medium, the optimal degradation temperature, pH value, and initial concentration of Atrazine are 25°C, 9.0, and 50 mg L^–1^, respectively, and the degradation rate of Atrazine by strain FH-1 reached 81.5% in 11 d of culture. The degrading process conforms to the kinetics equation of pesticide degradation. Among the metal ions tested, Zn^2+^ (0.2 mM) has the most significant effect of facilitation on the degradation of Atrazine. In the fluid medium with Zn^2+^, the degradation rate of Atrazine is increased to 72.5%, while the Cu^2+^ (0.2 mM) inhibits the degradation of Atrazine. The degradation products of Atrazine by strain FH-1 were identified as HEIT (2-hydroxyl-4-ethylamino-6-isopropylamino-1,3,5-triazine), MEET (2-hydroxyl-4,6-bis(ethylamino)-1,3,5-triazine), and AEEO (4,6-bis(ethylamino)-1,3,5-triazin-2(1H)-one) by HPLC-MS/MS. Three genes (*atzC*,* trzN*, and* trzD*) encoding for Atrazine degrading enzymes were identified by PCR and sequencing in strain FH-1. This study provides additional theoretical support for the application of strain FH-1 in bioremediation of fields polluted by Atrazine.

## 1. Introduction

Atrazine (2-chloro-4-ethylamino-6-isopropylamino-1,3,5-triazine) is one of the globally used triazine herbicides [1, 2], which is applied to control weeds in sorghum, corn, and sugarcane fields [3, 4]. Because of its high water solubility, long residual time, and being difficult to decompose, Atrazine can easily pollute the groundwater and cause damage to the succession crops and animals as well [5]. It is reported that 5% of Atrazine runs off with soil due to rainfall and irrigation and flows into aquatic ecosystem in the entire drainage basin [6]. Atrazine influences sex differentiation hormones in red claw crayfish during their early developmental stages and causes endocrine disorders, resulting in increased proportion of females in offspring [7]. Atrazine is also reported to affect the immune system of rabbits [8]. Furthermore, Atrazine has been reported to affect the formation of hemoglobins in humans [9]. Considering the wide application and high toxicity of Atrazine, it is of great importance to seek a practical way to rehabilitate the polluted environment caused by Atrazine.

At present, besides the underground phytoremediation [10] and the adsorption by nano-copper which induces the degradation of hydrocarbon radicals, microbial degradation is considered as the main, safe, and effective environmental bioremediation [11]. Microbial degradation of Atrazine is achieved by the modification of the amino sites on Atrazine [12]. Several species of fungi and bacteria have been identified to degrade Atrazine with high efficiency, including* Aspergillus niger* AN 400 [13],* Achromobacter* sp. [10],* Arthrobacter* sp. [14],* Delftia* sp. [15], and* Pseudomonas* sp. ZXY-1 [16]. The degradation pathway of Atrazine has been studied at molecular level. Atrazine is first catalyzed by the hydrolytic enzyme, encoded by genes* atzA* and* trzN*, to hydrolyze and dechlorinate to produce hydroxyatrazine; then, hydroxyatrazine hydrolytic enzyme (encoded by* atzB*) catalyzes hydroxyatrazine to transfer to N-isopropyl amide, which is hydrolyzed by N-isopropyl amide hydrolase (encoded by* atzC*) to generate cyanuric acid [17, 18]. Furthermore, some other intermediate degradation products have also been identified, such as 2-hydroxyl-4-ethylamino-6-isopropylamino-1,3,5-triazine (HEIT), 2-hydroxyl-4,6-bis(ethylamino)-1,3,5-triazine (MEET), and 4,6-bis(ethylamino)-1,3,5-triazin-2(1H)-one (AEEO) [19]. In this study, we isolated an Atrazine degrading bacterial strain FH-1 using enrichment culture from the soil with Atrazine applied over 10 years. We further identified this strain as* Klebsiella variicola* based on phylogenetic analysis of 16S rRNA gene sequences. This study provides additional theoretical support for the bioremediation of fields polluted by Atrazine. To our knowledge, the degradation of Atrazine by strain FH-1 has not been reported elsewhere.

## 2. Materials and Methods

### 2.1. Sample Collection

Soil samples were collected in the 0-20 cm top horizon layer at the Scientific Experimental Station, Jilin Agricultural University, China, where the fields have been applied with Atrazine for over 10 years. The soil has a pH value of 6.54 and organic matter of 18.57 g kg^–1^.

### 2.2. Chemicals and Media

Atrazine (purity: 97%) was purchased from TCI Development Co., Ltd. (Shanghai, China). All other chemicals consumed in our study were of analytical grade. Chemicals used for HPLC analysis were of HPLC grade. Degradation products of Atrazine (HEIT with purity of 98%, MEET with purity of 95%, and AEEO with purity of 96%) were purchased from Zhende Chemical (Shanghai) Co., Ltd. (Shanghai, China).

The mineral salt medium (MSM) used as the liquid enrichment medium contained 1.0 g NaCl, 1.5 g K_2_HPO_4_, 0.5 g KH_2_PO_4_, 0.2 g MgSO_4_·7H_2_O, and 1.0 g sucrose per liter. As the single nitrogen source, Atrazine was added from a stock solution (10 g L^–1^) in methanol to make the Atrazine mineral salt medium (AMSM). Lysogeny broth (LB) medium was made according to Yang et al. [20].

### 2.3. Enrichment and Isolation of Atrazine Degrading Bacteria

Soil sample (10 g) was added to a 250 mL conical flask containing 100 mL AMSM (Atrazine purity: 50 mg L^–1^), incubated aerobically at 30°C and shaken at 150 rpm for 7 days. Then, the enrichment culture (10 mL) was transferred to fresh AMSM containing Atrazine (100 mg L^–1^). To repeat 6 times of this process to obtain the increased concentration of Atrazine (600 mg L^–1^), the procedures of diluting the enrichment cultures and selecting of bacterial colonies were following those of Yang et al. [20]. A single bacterial strain FH-1 showing high biodegradation capacity was identified for further analysis.

### 2.4. Identification of Strain FH-1

Strain FH-1 was identified using the comparative analysis of 16S rRNA gene sequences. An EasyPure® Bacteria Genomic DNA Kit (TransGen Biotech Co., Ltd., Beijing, China) was used to extract the total genomic DNA of strain FH-1. PCR was applied to amplify the 16S rRNA gene using the universal forward and reserve primers of 27F (5′-AGAGTTTGATCCTGGCTCAG-3′) and 1492R (5′-GGTTACCTTGTTACGACTT-3′), respectively. Each PCR reaction contained Premix* Taq* 25 *μ*l, DNA template 500 ng, 1492R, and 27F primers 10 *μ*l, respectively, complemented by sterile water to a final volume of 50 *μ*l. The PCR cycle was set up as the following steps: (1) preheating (95°C for 5 min), (2) 35 cycles of denaturation (95°C for 30 s), annealing (56°C for 30 s), and extension (72°C for 1.5 min), and (3) a final extension (10 min at 72°C). An EasyPure Quick Gel Extraction Kit (TransGen Biotech Co., Ltd., Beijing, China) was used to purify the amplified PCR products with the 1% agarose gel electrophoresis. The purified PCR products were sequenced by Sangon Biotech Co., Ltd. (Shanghai, China). Phylogenetic and molecular evolutionary analyses were carried out using MEGA version 7.0 [21]. Phylogenetic trees were reconstructed using the neighbor-joining method [22] with bootstrap analysis of 1000 replicates.

### 2.5. Determination of Atrazine Content and Degradation Product

Atrazine was extracted three times from the soil samples with dichloromethane. The mixed extracts were concentrated and dried by evaporation. Blow-dried by N_2_, the constant volume was adjusted by mobile phase. HPLC (Agilent 1260) was used to measure the concentration of Atrazine from each sample. The wavelength was set to 222 nm on the UV detector and a reverse-phase column C_18_ (4.6 × 250 mm, 5 *μ*m) used a flow rate of 1.0 mL min^–1^ (methanol/water = 60/40, v/v), a column temperature of 30°C, and an injection volume of 10 *μ*L.

Liquid Chromatography-Mass Spectrometry (LC-MS/MS, UV-8030) was used to detect the Atrazine metabolites. The procedures of the chromatography analysis were performed by following those of previous studies [10, 23] with minor modifications. The chromatographic separations were made based on stationary phase with the analytical chromatographic column of XTerra MS C_18_ (3.9 mm × 100 mm × 3.5*μ*m, Waters, USA). The mobile phase was composed of a mixture of an aqueous solution containing 0.1% of formic acid and the acetonitrile under eluent (1:1, v/v). The flow rate of the mobile phase was 1.0 mL min^–1^.

### 2.6. Measurement of FH-1 Bacterial Growth Curve

Bacterial cells were cultured for 12 h in LB liquid medium containing Atrazine (50 mg L^–1^) and then centrifuged at 7000 rpm for 5 min. The cell precipitation was washed thrice with sterile water and resuspended in sterile water to achieve an absorbency value of 0.2. Then, 5 ml bacterial solution was added to 95 ml fluid medium. The growth of bacteria was examined every 4 h and the measuring time was 0-52 h by measuring the absorbance of the culture supernatant at 600 nm (OD_600_) using a UV2450-visible spectrophotometer (Shimadzu, Japan). HPLC (Agilent 1260) was used to measure the concentration of Atrazine.

### 2.7. Degradation Characteristics of FH-1 in Inorganic Salt Medium

#### 2.7.1. Effect of Temperature on Degradation of Atrazine by Strain FH-1

Atrazine was added to 100 ml basic inorganic salt medium (50 mg L^–1^) containing FH-1 bacterial solution (5%) with the pH value adjusted to 7.0. The incubation temperature was set to 20°C, 25°C, 30°C, 35°C, and 40°C, respectively, and shaken at 150 rpm, to measure the contents of Atrazine based on Qu et al. [24].

#### 2.7.2. Effect of Initial Concentration on Degradation of Atrazine by Strain FH-1

The initial concentration of Atrazine in medium with 5% FH-1 bacterial suspension added was adjusted to 10, 20, 50, 80, and 100 mg L^–1^, respectively, with pH value adjusted to 7. The incubation temperature was set to 30°C and shaken at 150 rpm to measure the contents of Atrazine.

#### 2.7.3. Effect of pH Value on Degradation of Atrazine by Strain FH-1

The pH values of the liquid medium with 5% FH-1 bacterial suspension added were adjusted to 5.0, 7.0, and 9.0, respectively, in treatment experiments, incubated at 30°C and shaken at 150 rpm. The bacterial growth and the concentration of Atrazine were measured at 3, 5, 7, 9, and 11 d of cultivation, respectively. All treatments were repeated thrice. Controls contained no bacterial suspension.

#### 2.7.4. Effect of Metal Ions on Degradation of Atrazine by Strain FH-1

Six common trace metal ion compounds (FeCl_3_, ZnCl_2_, CoCl_2_, NiSO_4_, CuSO_4_, and CaCl_2_) were selected to examine the effect of metal ions on the degradation of Atrazine by strain FH-1. These compounds were first weighed and dissolved in sterile water. The concentration of trace metal ions in the inorganic salt medium was set to 0.2 mM based on a previous study in order for bacteria to maintain their normal growth [25]. The pH value was adjusted to 7.0 in the inorganic salt medium with both FH-1 bacterial suspension (5%) and Atrazine (50 mg L^–1^) added. The incubation temperature was set to 30°C and shaken at 150 rpm. In 5 days of cultivation, HPLC (Agilent 1260) was used to measure the contents of Atrazine. Both treatment experiments and controls were each repeated three times.

### 2.8. Analysis of Degradation Products

FH-1 bacterial suspension (5%) was added to the 100 mL basic salt medium containing Atrazine (50 mg L^–1^) with pH value adjusted to 9.0 and cultured in a shaking bed at 25°C and 150 rpm. The products were determined by HPLC-MS/MS (UV-8030, Japan) on 3, 5, 7, 9, and 11 d, respectively. Each sample was first filtered by a 0.45 *μ*m nylon filter prior to the measurement by HPLC-MS/MS. Each treatment was repeated three times and the controls contained no bacterial suspension.

### 2.9. Identification of Atrazine Degrading Genes

PCR was performed using available primers [26–29] to determine the presence of eight Atrazine degrading genes, including* atzA*,* atzB*,* atzC*,* atzD*,* atzE*,* atzF*,* trzD*, and* trzN*, in strain FH-1 (Table 1). The total genomic DNA in FH-1 was used as the PCR template. PCR reactions were conducted using the following thermocycler program: preheating 5 min at 95°C, 35 cycles of denaturation 1 min at 94°C, annealing for 1 min at the optimal temperature, and extension 2 min at 72°C, and then an additional extension 10 min cycle at 72°C. PCR products were examined using 1% agarose gel electrophoresis.

### 2.10. Statistical Analysis

Results of the measurements were given as the average±standard deviation of each treatment. Correlation analysis and the one-way analysis of variance (ANOVA) were performed using SPSS (version 21.0) to evaluate the statistical difference among all of the groups based on a* p* value of 0.05.

## 3. Results

### 3.1. Separation and Identification of Strain FH-1

Strain FH-1, currently stored in the China Center for Type Culture Collection (http://www.cctcc.org/; M2018334), with high degradation ability of Atrazine, was selected for further experiments. Morphological observations revealed that the colonies of strain FH-1 were viscid and smooth, spherical or roughly spherical in shape, with soft and milky texture and slight luster. Strain FH-1 grew rapidly on LB solid plates. Strain FH-1 is Gram-negative, and its immotile cells are atrichia and short rod in shape with rough surface.

**Figure 1:**
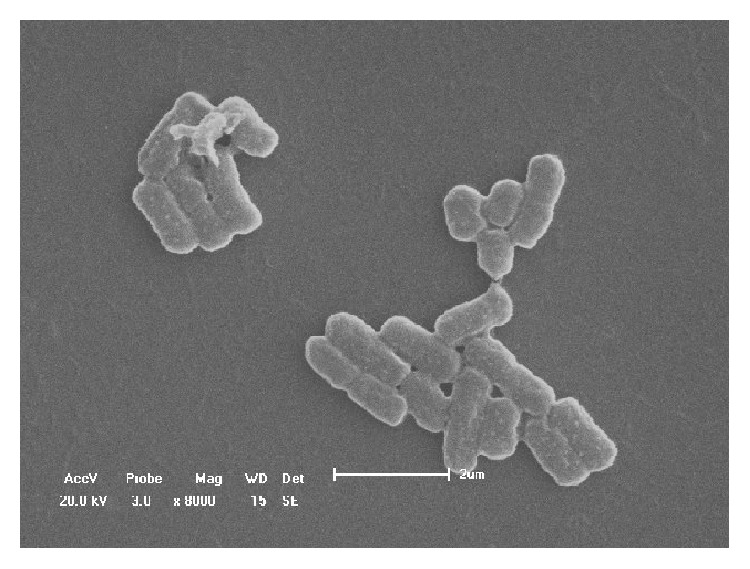
Scanning electron micrograph of strain FH-1.

The morphology of the strain FH-1 was also observed by scanning electron microscope (Figure 1). Strain FH-1 was a short, flat, coarse bacillus with a size of 0.5–0.8 × 1–2 *μ*m. The cells either were alone or were arranged in double or short chains, without spores and flagella but a thick capsule. The physiological and biochemical characteristics of strain FH-1 were tested. Results of physiological and biochemical tests showed that strain FH-1 was facultative and anaerobic revealed by the aerobic respiration test and there were two types of metabolic pathways, i.e., aerobic respiration and fermentation (revealed by the positive results of glucose fermentation, citric fermentation, sucrose fermentation, cellobiose fermentation, and maltose fermentation), with a positive result of the VP test and negative results of gelatin liquefaction (Table 2).

**Figure 2:**
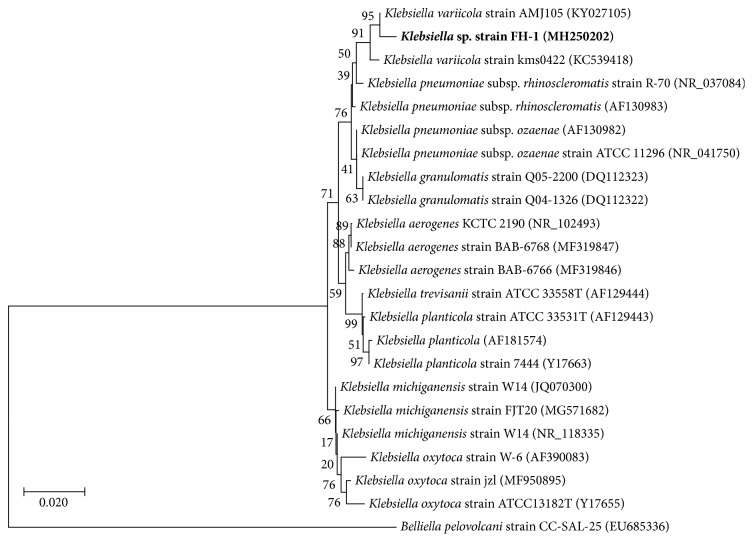
Phylogenetic tree based on DNA sequences of 16S rRNA gene using neighbor-joining method.* Klebsiella* sp. strain FH-1 is shown in bold.* Belliella pelovolcani* is used as the outgroup. GenBank accession numbers are given in parentheses following the species names. Bootstrap values based on 1000 replicates are given next to the branches. The bar indicates 0.020 base difference per nucleotide position.

The strain FH-1 was 1442 bp in length (stored in GenBank with an accession number of MH250202) and was used to conduct the BLAST (https://blast.ncbi.nlm.nih.gov/Blast.cgi) search in the NCBI (https://blast.ncbi.nlm.nih.gov/Blast.cgi) database. The results of genetic distance showed that the strain FH-1 was closely related to* Klebsiella*, showing over 99% homology of 16S rDNA between strain FH-1 and other species in* Klebsiella*. A phylogenetic tree based on16S rDNA sequences was reconstructed by neighbor-joining method (Figure 2), showing that strain FH-1 was closely related to two strains of* Klebsiella variicola* (strains kms0422 and AMJ105). Other closely related species included* K. pneumoniae*,* K. granulomatis*,* K. aerogenes*,* K. planticola*,* K. oxytoca*, and* K. michiganensis*. Most of these species were revealed as monophyletic groups. Based on these results, strain FH-1 is recognized as* Klebsiella variicola*.

### 3.2. Relationship between the Growth of Strain FH-1 and the Degradation of Atrazine

**Figure 3:**
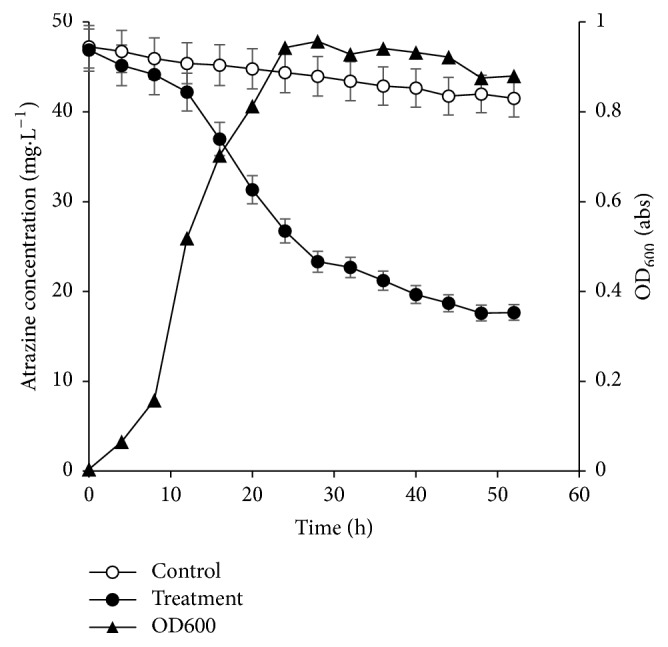
Bacterial growth curve of strain FH-1 and degradation of Atrazine by strain FH-1.

The growth curve of strain FH-1 cultured in LB medium showed that strain FH-1 entered the logarithmic phase of growth at 12-16 h but with low degradation rate of Atrazine of 5.24 mg kg^–1^ (Figure 3). The degradation of Atrazine was evidently accelerated by the presence of strain FH-1. OD_600_ value reached its highest (0.943) at 24 h, and degradation rate was up to 54%. After 44 h, OD_600_ values started to decrease and FH-1 entered a period of decay. These results showed that the strain FH-1 grows well in the presence of Atrazine (50 mg L^–1^) and its growth cycle was relatively short. Under these laboratory conditions, Atrazine is removed with high efficiency by strain FH-1 (81.5%), which meets the requirements of degradation and bioremediation. Therefore, the experiments did not go beyond 11 days.

### 3.3. Degradation Characteristics of Strain FH-1

Table 3 showed the kinetic degradation equations of Atrazine with various initial concentrations by strain FH-1 cultured under 30°C with pH value of 7.0. The degrading process conforms to the kinetic equation, which is given as lnC = −Kt + A, where C is the concentration of Atrazine, t is the time interval, K is the constant of degradation rate, and A is the constant. These results showed that the 50 mg L^–1^ is the optimal degrading concentration for Atrazine with a degradation rate of 3.08 mg l^–1^ d^–1^ and a half-life of 4.2 days.

**Figure 4:**
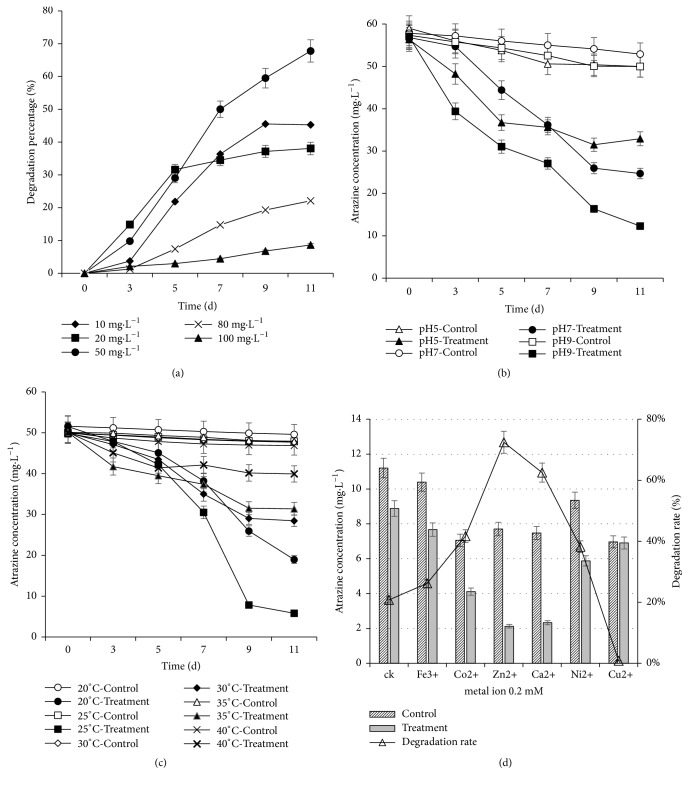
Effects of initial concentration of Atrazine (a), pH value (b), temperature (c), and metal ion (d) on the degradation of Atrazine by strain FH-1 cultured in 5 days.

The degradation rates of Atrazine at various starting concentrations by strain FH-1 are shown in Figure 4(a). When the concentrations of Atrazine were 10 mg L^–1^ and 20 mg L^–1^, the degradation rates of Atrazine reached 45.3% and 38.1%, respectively, in 11 days. When the concentrations of Atrazine were increased to 50 mg L^–1^, the degradation rate of Atrazine increased to 67.8%. The degradation rates decreased with the increase of Atrazine concentration larger than 50 mg L^–1^. When the concentrations of Atrazine were increased to 80 mg L^–1^ and 100 mg L^–1^, the degradation rates of Atrazine decreased to 22.1% and 8.6%, respectively (Figure 4(a)). The degradation of Atrazine by strain FH-1 under pH values of 5.0, 7.0, and 9.0 for 3, 5, 7, 9, and 11 d, respectively, was shown in Figure 4(b). When pH values were 5.0 and 7.0, the degradation rates were 41.6% and 56.6% in 11 days of culture, respectively. The optimal pH value was 9.0 and the degradation rate of Atrazine reached 78.4% after 11 days of culture. The optimal temperature for Atrazine degradation was 25°C (Figure 4(c)) when the degradation rate of Atrazine reached 88.3% in 11 days of culture. These results showed that the strain FH-1 demonstrated great potential for the bioremediation of contaminated sites by Atrazine.

The effects of various metal ions (0.2 mM) on the degradation of Atrazine were shown in Figure 4(d). In 5 days of culture, most of the tested ions accelerate the degradation of Atrazine by strain FH-1, except for Cu^2+^, which inhibited the degradation of Atrazine by FH-1 from 20.8% (in controls) to 0.8%. The promoting effect of Zn^2+^ on the degradation of Atrazine was the most evident, which increased the degradation of Atrazine from 20.8% (in controls) to 72.5%. These results showed that the degradation of Atrazine by strain FH-1 was improved by adding appropriate metal ions to the inorganic salt medium.

### 3.4. Degradation Products of Atrazine

**Figure 5:**
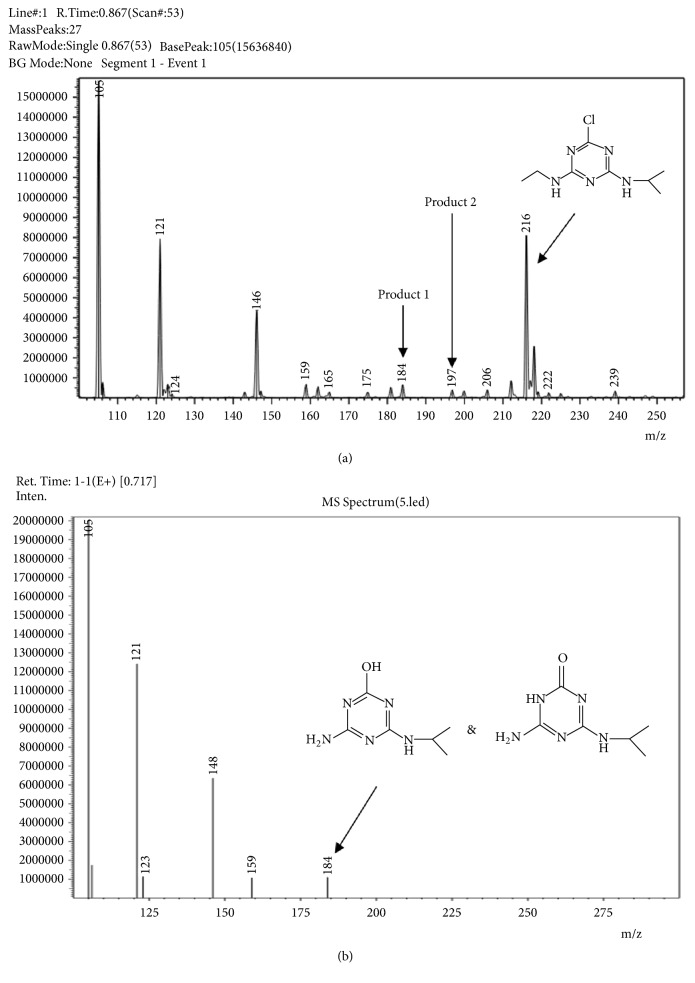
Degrading metabolites of Atrazine determined by HPLC-MS/MS with a full scan (a) and a single ion monitor (b). Product 1 is AEEO and MEET (target fragment-ions 184) (a, b) with chemical structures given in (b), AEEO on right and MEET on left. Product 2 is HEIT (target fragment-ions 197) (a). Chemical structure of Atrazine (target fragment-ions 216) is given in (a).

After the Atrazine was degraded by strain FH-1 in inorganic salt medium for 5 days, the degradation products were analyzed by HPLC-MS/MS (UV-8030). The three degradation products of Atrazine were HEIT, AEEO, and MEET, with molecular masses of 197, 183, and 183, respectively, and were shown in Figure 5(a). The presence of other peaks in Figure 5 is probably due to the substances in the medium, which cannot be determined due to the lack of standards in these experiments. Because both AEEO and MEET can bind to H^+^ easily, therefore, the molecular mass of both AEEO and MEET was revealed as 184, based on mass spectrometry (Figure 5(b)). Both AEEO and MEET have the same molecular mass and therefore are identified on the same peak (Figure 5). The results also showed that HEIT and Atrazine did not bind with H^+^. The characteristics of the HPLC-MS/MS analyses of Atrazine and its degradation products were summarized in Table 4. All substances examined showed high average recovery rates and low relative standard deviation.

### 3.5. Degradation Genes in Strain FH-1

**Figure 6:**
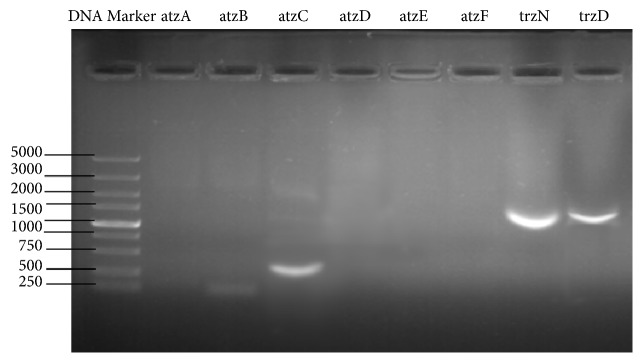
Agarose gel electrophoresis (1%) of PCR products of Atrazine degrading genes in strain FH-1.

PCR analysis was carried out to identify the Atrazine degrading genes* atzC* (624 bp in length),* trzN* (1110 bp), and* trzD* (1145 bp) in strain FH-1 (Figure 6). The DNA sequences of these genes are stored in GenBank:* atzC* (MK037388),* trzN* (MK037389), and* trzD* (MK037390). Specifically,* atzC* encodes the N-isopropyl amide hydrolase,* trzN* encodes Dechlorination hydrolase, and* trzD* encodes Cyanuric acid hydrolase. Although it is noted that gene* atzB* might be present with a weak band of ~250 bp (Figure 6), we failed to isolate this product. In comparison to the length (~500 bp) of gene* atzB* reported in GenBank, this band of ~250 bp may not be the DNA fragment of gene* atzB* in strain FH-1.

## 4. Discussion

### 4.1. Relationship between the Growth of Strain FH-1 and the Degradation of Atrazine

The growth rate of strain FH-1 in LB medium was slow from 0 to 12 hours, indicating a period of adaptation to the environment. Consequently, the degradation of Atrazine was not evident during this period (Figure 3). When the growth of strain FH-1 entered logarithmic phase from 12 to 16 hours, the Atrazine was consumed for growth and reproduction by bacteria, causing the degradation rate of Atrazine by FH-1 to accelerate. In 28 h of growth, strain FH-1 entered the stable and decay stages, which may be related to the accumulation of metabolites, causing some bacteria to die or even autolysis and the growth of FH-1 tended to be flat or even decreased, while the degradation rate of Atrazine by strain FH-1 tended to be moderate again. These results further demonstrated that Atrazine may not be the optimal nitrogen source for the bacterial growth, while the degradation rate of Atrazine by strain FH-1 is increased with the lack of the optimal nitrogen source. In the selection of base inorganic salts, no nitrogen-containing substance was added. Strain FH-1 could grow in this environment with Atrazine added, indicating that Atrazine provided the only nitrogen source.

### 4.2. Degradation Characteristics of Atrazine by FH-1

In MSM, the half-life of Atrazine degradation was shortened and the degradation rate of Atrazine was increased by adding strain FH-1 (Table 3, Figure 4(a)). The degradation rate of Atrazine is 3.08 mg l^–1^ d^–1^ when the optimal starting concentration of Atrazine is 50 mg L^–1^ in MSM with the half-life of 4.2 d. When the concentrations of Atrazine were lower than 50 mg L^–1^ (e.g., 10 mg L^–1^ and 20 mg L^–1^) or higher than 50 mg L^–1^ (e.g., 80 mg L^–1^ and 100 mg L^–1^), the degradation rates were both slower than those under 50 mg L^–1^. This is probably because the low concentration of Atrazine cannot provide enough nitrogen source to make the bacteria grow normally, while the high concentration of Atrazine inhibits the growth of strain FH-1, consequently decreasing the degradation ability of strain FH-1 on Atrazine. Similar results were also reviewed previously [30]. Together with the optimal pH value (9.0) and temperature (25°C) (Figures 4(b) and 4(c)) for degradation of Atrazine, these results indicated that it is practically feasible to remove the Atrazine residuals in nature by using the strain FH-1, which degrades Atrazine with high efficiency under the laboratory conditions. For example, the degradation rate of Atrazine is the highest at 25°C, indicating that strain FH-1 is suitable for growth and reproduction at 25°C. When the temperature was higher than 40°C, the bacteria can hardly grow, suggesting that the degradation rate of Atrazine was affected by the temperature by mainly increasing the concentration of bacterial cells, similar to the results as previously reported [31].

The optimal degrading concentration for Atrazine by strain FH-1 (50 mg L^–1^) is much higher than that of* Aspergillus niger* AN 400 (30 mg L^–1^) [13], while the latter is more resistant to drugs and more dependent on glucose, demonstrated by the low degradation rate of Atrazine (40%) without the presence of glucose or under low content of glucose [13]. This suggests that strain FH-1 is more adaptive in a changing environment. Furthermore,* Pseudomonas* sp. ZXY-1 [16] showed similar degrading capability of Atrazine as that of strain FH-1, with comparable optimal temperature, pH value, inoculation, and initial concentration of 30.71°C, 7.14, 4.23% (v/v), and 157.1 mg kg^–1^, respectively. However,* Pseudomonas* sp. ZXY-1 showed decreased degradation rate under low temperature or more alkaline environments. Therefore, it is practically imperative practical to study the degrading mechanism of Atrazine by strain FH-1. In Northeastern China, the general application of 1.7-2.6 kg hectare^–1^ of the herbicide in the corn field generates the accumulation of Atrazine of about 2.89-3.81 mg kg^–1^ and may be higher due to the overdose of the herbicide [14]. This concentration of Atrazine in the field is much lower than that of Atrazine (50 mg L^–1^) in the liquid medium in our study.

### 4.3. Effect of Metal Ions on the Degradation of Atrazine by FH-1

When trace metal ions were added to inorganic salt medium, the degradation of Atrazine was accelerated by most of the ions tested (Figure 4(d)), except for Cu^2+^, which inhibited the growth of bacteria, because most bacteria needed trace metal ions to provide energy. Specifically, when the electrons are lost in the process of degradation, the energy generated is provided for bacterial degradation [32]. The inhibitory effect of Cu^2+^ on bacteria may be due to the denaturation of enzymes degrading Atrazine caused by Cu^2+^ or because Cu^2+^ can block the hydrogen bond of bacterial protein and inhibit its growth and metabolism [33]. The antibacterial mechanism of Cu^2+^ has also been further explored from other two perspectives. First, the dysfunction or destruction of the internal components of the microorganisms is caused by the contact by Cu^2+^. Second, during the photocatalytic reaction, Cu^2+^ functions as a catalytically active center under light to activate oxygen in water and air to generate hydroxyl radicals and reactive oxygen ions, quickly destroying the reproduction of bacteria and causing bacteria to die [34]. The characterization of the degradation of Atrazine by strain FH-1 showed that the growth rate of bacteria was low and the degradation rate of Atrazine was also low during the first 3 days of culture (Figure 4(a)), which may be due to the adaptation of bacteria to the new environment [35]. It has been demonstrated that the degradation rate of Atrazine in alkaline environment was higher than that in neutral or acidic environments [36]. It is possible that strain FH-1 was isolated from the soil with high alkalinity and the degrading activity in alkaline condition was higher than that in neutral and acidic environments; ultimately the bacteria could survive more easily. Consequently, the degradation rate of Atrazine was increased due to the presence of a large number of bacteria.

The promoting effect of Zn^2+^ on the degradation of Atrazine was the most evident (Figure 4(d)). As a cofactor of alcohol dehydrogenase, Zn^2+^ is widely found in many hydrolases involved in the metabolism of sugars, proteins, and nucleic acids, which are affected significantly by the amount of Zn^2+^. The moderate amount of Zn^2+^ may promote the growth of bacteria and the production of hydrolase, consequently increasing the total amount of bacteria of strain FH-1 in the degradation process. This is observed in Figure 4(d), showing that, in the presence of Zn^2+^, the hydrolysis is strengthened even in the control group with the presence of Zn^2+^. The mechanism of Zn^2+^ improving bacterial degradation of organic matter has been reported [37]. Without water, Zn^2+^ damages the C-Cl bond in the organic compounds, causing the organic matter to undergo partial hydrolysis and improving the catalytic effect on degradation by the bacteria. In water, the ability to catalyze the hydrolysis by Zn^2+^ is enhanced. The mechanism of this enhancement is as follows. The OH- replaces the Cl- in the C-Cl bond of the organic compound to form a C-OH functional group, consequently strengthening the damage of the C-Cl bond to form C=C and improving the ability of Zn^2+^ to catalyze hydrolysis [37]. It is important to further study the mechanisms affecting the degradation of Atrazine by both ions Cu^2+^ and Zn^2+^. This research is currently undergoing but beyond the scope of current study which is to characterize the degradation of Atrazine by the novel strain of* Klebsiella variicola* strain FH-1.

### 4.4. Atrazine Degrading Genes in Strain FH-1

Three Atrazine degrading genes (*atzC*,* trzN*, and* trzD*) were identified in strain FH-1 (Figure 6). The functions of these genes in the degradation of Atrazine have been extensively studied. In general, the first step in the degradation of Atrazine is to produce hydroxyatrazine by water dechlorination, which is catalyzed by chlorohydrolase (encoded by* atzA* or* trzN*). Secondly, hydroxyatrazine ethylaminohydrolase (encoded by* atzB*) catalyzes the conversion of hydroxyatrazine to N-isopropyl amide. Finally, N-isopropyl amide is hydrolyzed by N-isopropyl amide hydrolase (encoded by* atzC*) to produce cyanuric acid [17, 18, 38], which is further degraded to generate Biuret and ultimately NH_3_ and CO_2_ by the enzymes encoded by either* atzD* or* trzD* [18, 39]. Apparently, the three Atrazine degrading genes (*atzC*,* trzN*, and* trzD*) identified in strain FH-1 do not function together and therefore are not clustered together in the same operon. Furthermore, the PCR experiments applied to amplify these three Atrazine degrading genes were based on the genomic DNA, not plasmid DNA, indicating that these three genes are located on the chromosome but not plasmid of strain FH-1. Further genomic sequencing currently undergoing is necessary to reveal the existence of plasmids in strain FH-1. Studies showed that* Pseudomonas* sp. [10] contains a total of 6 Atrazine degrading genes (*atzA*,* atzB*,* atzC, atzD*,* atzE*, and* atzF*), while* Arthrobacter* sp. [14] has only three (*atzA*,* atzB*, and* atzC*) and strain FH-1 has three (*atzC*,* trzN*, and* trzD*). It is clear that the species with three Atrazine degrading genes identified are capable of degrading Atrazine. However, it is still possible that these species may contain different genes encoding different Atrazine degrading enzymes. Based on the comparative analysis of DNA sequences of these genes and functional prediction of these genes, it is suggested that* atzC* is likely to encode the proteins which function on the diazo bonds of the aromatic compounds;* trzN *is likely to encode the aerobic respiration two-component sensor histidine kinase, while* trzD* is likely to encode acyltransferase. The functional mechanisms of these genes would be worthy of further investigation. PCR experiments showed that the genetic potential for the degradation of Atrazine by FH-1 is similar to that of strain TC1 [17, 40]. BLAST analyses of these Atrazine-degrading genes in strain FH-1 using the NCBI database have revealed many homologous gene sequences in other bacterial species. For example, the* atzC* in strain FH-1 is closely related to the* atzC* in* Bacterium* sp. TN196,* Arthrobacter* sp. DNS10, and* Comamona*s sp. A2. Gene* atzC* encodes the enzyme atzC, which further catalyzes N-isopropylammelide to yield cyanuric acid; a similar process is found in several other species, including* Sinorhizobium* sp. NEA-B and* Polaromonas* sp. NEA-C [41],* Arthrobacter* sp. AD26 [42], and* Nocardioides* sp. strains EAA-3 and EAA-4 [43]. Furthermore, the* trzN* in strain FH-1 is closely related to the gene* trzN* in* Bacterium* sp. TN198 and* Arthrobacter* sp. DNS10. The isotope fractionation occurs during the degradation of Atrazine and the function of* trzN* is similar to that of* atzA* having similar biochemical conversion mechanisms of conversion, both catalyzing the first reaction of the Atrazine degradation [44]. It is noted that the gene* atzA* is located on the plasmid of* Pseudomonas* sp. [45], while the gene* trzN* of FH-1 strain with high homology to* atzA* is located on the chromosome of strain FH-1. Moreover, the* trzD* in strain FH-1 finds its homolog in* Chelatobacter heintzii* strain Cit1 with a 55% similarity. Further studies based on comprehensive sampling of bacterial species are needed to infer the evolutionary histories of these genes and to investigate the mechanisms, for example, horizontal gene transfer, of obtaining the function of degrading Atrazine among different bacterial species. The presence of these Atrazine degrading genes in strain FH-1 provides strong theoretical support for the application of strain FH-1 in bioremediation of fields contaminated by Atrazine. It is noted that other Atrazine degrading genes may be present but were not amplified due to the possible variations of sequences between FH-1 and reference strains. Further experiments are necessary to verify the presence and functions of these enzymes encoded by the genes identified in our study.

## 5. Conclusions

Results of our studies demonstrate that the* Klebsiella variicola *strain FH-1 is capable of effectively degrading Atrazine. The degradation of Atrazine by strain FH-1 is inhibited or facilitated by ions Cu^2+^ or Zn^2+^, respectively. The degradation products of Atrazine by strain FH-1 have been identified as HEIT, MEET, and AEEO by HPLC-MS/MS. Three genes (*atzC*,* trzN*, and* trzD*) encoding for Atrazine degrading enzymes have also been identified by PCR and sequencing in strain FH-1. Further experiments are necessary to confirm the presence and functions of the enzymes encoded by these genes identified in this study. Our study provides theoretical support for the application of strain FH-1 in bioremediation of fields polluted by Atrazine.

## Figures and Tables

**Table 1:** Primers (F for forward and R for reverse) used for PCR amplification of Atrazine-degrading genes.

Gene	Primer	Nucleotide sequence (5′-3′)	Annealing temperature (°C)	Reference
*atzA*	atzA-R	TGAAGCGTCCACATTACC	50	[26]
	atzA-F	CCATGTGAACCAGATCCT		
*atzB*	atzB-R	CTCTCCCGCATGGCATCGGG	63	[26]
	atzB-F	TCACCGGGGATGTCGCGGGC		
*atzC*	atzC-R	GTACCATATCACCGTTTGCCA	55	[26]
	atzC-F	GCTCACATGCAGGTACTCCA		
*atzD*	atzD-R	TGTCGGAGTCACTTAGCA	50	[29]
	atzD-F	ACGCTCAGATAACGGAGA		
*atzE*	atzE-R	GGAGACCGGCTGAGTGAGA	50	[29]
	atzE-F	TACGCGGTAAAGAATCTGTT		
*atzF*	atzF-R	CGATCGCCCCATCTTCGAAC	55	[29]
	atzF-F	CGATCGGAAAAACGAACCTC		
*trzN*	trzN-R	GATTCGAACCATTCCAAACG	55	[28]
	trzN-F	CACCAGCACCTGTACGAAGG		
*trzD*	trzD-R	GTTACGAACCTCACCGTC	50	[27]
	trzD-F	TACGCGGTAAAGAATCTGTT		

**Table 2:** Characteristics of the physiological and biochemical tests of strain FH-1. Symbol “+” indicates positive and “–” negative.

Test	Result
Oxidase	–
Anaerobic growth	+
VP test	+
Gelatin liquefaction	–
Growth at 37°C	+
Glucose fermentation	+
Citric fermentation	+
Sucrose fermentation	+
Cellobiose fermentation	+
Maltose fermentation	+

**Table 3:** Kinetic degradation equations of Atrazine with various initials concentrations by strain FH-1.

Initial concentration	Degradation rate (mg l^–1^ d^–1^)	Kinetic equation	Correlation coefficient (R^2^)	Degradation half-life (days)
100 mg L^–1^	0.78	lnC = −0.018t + 4.62	0.984	38.7
80 mg L^–1^	1.62	lnC = −0.058t + 4.46	0.929	13.2
50 mg L^–1^	3.08	lnC = −0.241t + 4.23	0.948	4.2
20 mg L^–1^	0.76	lnC = −0.093t + 3.0	0.808	7.5
10 mg L^–1^	0.46	lnC = −0.141t + 2.6	0.950	6.1

**Table 4:** The characteristics of the HPLC-MS/MS analyses of Atrazine and its metabolites.

Chemicals	Limit of detection (mg kg^–1^)	Average recovery rate (%)	Relative standard deviation (%)
Atrazine	0.02	93.5–106.4	0.09–1.07
HEIT	0.05	70–107	1.58–11.13
MEET	0.05	70.2–105	6.85–12.77
AEEO	0.05	70.6–109.1	0.65–5.18

## Data Availability

The data used to support the findings of this study are available from the corresponding authors upon request.
